# *Klebsiella pneumoniae* Isolates from Meningitis: Epidemiology, Virulence and Antibiotic Resistance

**DOI:** 10.1038/s41598-017-06878-6

**Published:** 2017-07-26

**Authors:** Yee-Huang Ku, Yin-Ching Chuang, Chi-Chung Chen, Mei-Feng Lee, Yan-Chang Yang, Hung-Jen Tang, Wen-Liang Yu

**Affiliations:** 1 Division of Infectious Disease, Department of Internal Medicine, Chi Mei Medical Center-Liouying, Tainan, Taiwan; 2 Department of Medical research, Chi-Mei Medical Center, Tainan, Taiwan; 3 Division of Infectious Disease, Department of Internal Medicine, Chi Mei Hospital, Tainan, Taiwan; 4 Department of Health and Nutrition, Chia Nan University of Pharmacy and Science, Tainan, Taiwan; 5 Department of Intensive Care Medicine, Chi-Mei Medical Center, Tainan, Taiwan; 6 Department of Medicine, School of Medicine, College of Medicine, Taipei Medical University, Taipei, Taiwan

## Abstract

*Klebsiella pneumoniae* (KP) resistance to broad-spectrum cephalosporin (BSC) in meningitis is important because of limited therapeutic options. To investigate the antibiotic resistance, virulence and epidemiology of KP in meningitis, we conducted a retrospective study for 33 non-metastatic isolates, including primary meningitis (*n* = 20) and post-craniotomy meningitis (*n* = 13) collected from 1999 to 2013. BSC resistance was found in 9 (27.3%) isolates, all from post-craniotomy meningitis, harboring *bla*_SHV-5_ (*n* = 6), *bla*_CMY-2_ (*n* = 2), *bla*_DHA-1_ (*n* = 2), and *bla*_TEM-1B_ (*n* = 1). Positive virulence factors were hypermucoviscosity (*n* = 22), larger bacterial size (*n* = 24), virulent capsule serotypes (*n* = 24, K2, 11; K1, 5; K57, 3; K5, 2; K20, 2 and K54, 1), *rmpA* (*n* = 23), *rmpA*_*2*_ (*n* = 20), aerobactin gene (*n* = 22) and high-grade serum resistance (*n* = 23, 69.7%). Higher mouse lethality (LD_50_ < 10^6^) was found in 16 isolates (48.5%). Post-craniotomy isolates were significantly less virulent than primary meningitis isolates, except for similar serum resistance capability. The pulsotype and sequence typing (ST) results were diverse. A minor cluster with pulsotype C and ST23 (*n* = 5) was ide*n*tified in primary meningitis isolates. In conclusion, virulence factors and BSC resistance corresponded to about 70% and 30% of KP meningitis isolates respectively. BSC remains appropriate for treating primary meningitis, whereas meropenem is indicated for post-craniotomy meningitis empirically.

## Introduction

*Klebsiella pneumoniae* causes different types of community-acquired and healthcare-associated infections, including pneumonia, bloodstream infection, surgical site infections, liver abscess and meningitis^1–4^. The clinical spectrum of *K*. *pneumoniae* meningitis can be categorized into 3 distinct forms: first, metastatic meningitis, particularly from the distant liver abscesses^1^; second, post-craniotomy meningitis following neurosurgical procedures for brain lesions or head injury; and third, primary or spontaneous meningitis, usually among elderly patients or those with underlying immunocompromised conditions^5^.

In Taiwan, *K*. *pneumoniae* has remained the leading causative pathogen with prevalence rates about 25% to 40% among adult patients with bacterial meningitis^6–10^. Besides, *K*. *pneumoniae* was responsible for 50% of severe meningitis in adult patients who required intensive care and up to 68% among a subpopulation of adult diabetic patients^11, 12^, with high mortality rates ranging from 48.5% to 66%^5, 13^.

The pathogenesis of *K*. *pneumoniae* for being capable of invasion into the central nervous system is not well elucidated. The meningeal metastatic isolates frequently arising from the liver abscess have been recognized as “hypervirulent” *K*. *pneumoniae* (hvKP), which is commonly hypermucoviscous on blood agar plates^2, 4^. Distinct from hvKP, less virulent “classic” *K*. *pneumoniae* (cKP) that does not exhibit the hypermucoviscosity (HV) phenotype usually causes various infections in hospitals^14^. We previously reported that the *rmpA* (regulator of mucoid phenotype) gene was highly prevalent among both primary meningitis and liver abscess isolates^15^. Nonetheless, the detailed profiles of virulence, virulence determinant factors and relationship with antibiotic resistance of *K*. *pneumoniae* from non-metastatic meningitis isolates have not been fully characterized. We hypothesized that post-craniotomy meningitis could be caused by cKP through disruption of the central nervous system barrier, whereas primary meningitis might commonly be caused by hvKP.

Therefore, we aimed to investigate the molecular epidemiology and representative virulence factors in terms of capsule sizes, capsule serotypes, HV phenotype, serum resistance, pathogenic genes, including plasmid-borne *rmpA*, *rmpA2*, aerobactin gene and chromosomal *rmpA* (c-*rmpA*), *kfu* (responsible for an iron uptake system) and *allS* (associated with allantoin metabolism), as well as mouse lethality capability for *K*. *pneumoniae* strains isolated from cerebrospinal fluid (CSF) among adult patients without liver or distant abscesses. Antibiotic resistance profiles and virulence factors between non-metastatic *K*. *pneumoniae* isolates of primary meningitis and post-craniotomy meningitis were compared.

## Materials and Methods

### Bacterial isolates

We previously collected 33 *K*. *pneumoniae* isolates from CSF of non-metastatic meningitis patients without liver or distant abscess, including isolates from Taipei Veterans General Hospital in Taipei City, northern Taiwan, 2003–2006 (*n* = 7); National Cheng-Kung University Hospital in Tainan City, southern Taiwan, 1999–2007 (*n* = 10); and Chi-Mei Medical Center in Tainan City, southern Taiwan, 2005–2013 (*n* = 16). The isolates included primary meningitis (*n* = 20) and post-craniotomy meningitis (*n* = 13). These isolates were obtained as part of routine care on clinical indication.

### Antimicrobial susceptibility testing

The minimum inhibitory concentrations (MICs) of antimicrobial agents were determined by using the agar dilution method^16^. The tested compounds included ampicillin, aztreonam, cefotaxime, ceftazidime, cefepime, piperacillin-tazobactam, meropenem, ciprofloxacin, colistin sulfate (Sigma Chemical Company, St. Louis, MO, USA) and tigecycline (Wyeth, Puerto Rico).

According to the British Society for Antimicrobial Chemotherapy Standing Committee for Antimicrobial Susceptibility Testing (Version 14.0, 2015), the susceptible MIC breakpoint for colistin against Enterobacteriaceae is ≦2 μg/mL and it is considered resistant if MIC > 2 μg/mL. The breakpoints for tigecycline against Enterobacteriaceae are ≦1μ g/mL as susceptible and >2 μg/mL as resistant.

### Phenotypic detection of extended-spectrum β-lactamase (ESBL)

ESBL double-disk synergy test was performed with disks containing ceftazidime or cefepime with clavulanic acid on Mueller-Hinton agar plates, and the results were interpreted as described previously^17^. Control experiments were assured by testing *E*. *coli* ATCC 25922 and *K*. *pneumoniae* 700603.

### DNA manipulation and PCR amplification

Genomic DNA was extracted using QIAamp DNA Mini Kit (Qiagen, Hilden, Germany). Plasmid DNA was also extracted by QIAprep Spin Miniprep (Qiagen, Hilden, Germany). PCR amplifications were performed by using specific primers as previous described.

### PCR detection and sequencing of antibiotic resistance genes

Plasmid DNA as templates, PCR was used to amplify the ESBL genes (*bla*_TEM_, *bla*_SHV_, *bla*_CTX-M_), *ampC* genes (*bla*_DHA-1_ and *bla*_CMY-2_) and to screen the representative carbapenemase gene (*bla*_KPC-2_) using specific primers as previously published^18^. Amplicons of β-lactamase genes were purified with PCR clean-up kits (Roche Diagnostics, GmbH, Penzberg, Germany) and were sequenced on an ABI PRISM 3730 sequencer analyzer (Applied Biosystems, Foster City, CA, USA).

### PCR detection of virulence factors

Genomic DNA as templates, PCR was used to amplify the K capsule serotype-specific genes (including 6 liver abscess-associated capsule serotypes K1, K2, K5, K20, K54, and K57), c-*rmpA*, *kfu* and *allS* genes. Plasmid DNA as templates, PCR was used to amplify the *rmpA*, *rmpA2* and aerobactin gene. Specific primers were used as previously described^19–23^.

### Phenotypic detection of virulence factors with HV phenotype and capsule size

The HV phenotype was defined positive as a viscous string of >5 mm of the colony on blood agar plate^24^. We measured bacterial size to represent capsule size of the bacteria. The microscopic images of bacterial size in short transverse diameter randomly counted from 100 bacterial cells per single isolate were analyzed by using the image analysis software cellSens standard version 1.8 (Olympus Optical Co. Ltd., Tokyo, Japan).

### Phenotypic detection of virulence factor with serum resistance

The susceptibility of the *K*. *pneumoniae* isolates to human serum was analyzed as described previously^25^. In brief, twenty-five microliters of the bacterial suspension (about 2 × 10^6^ CFU) were mixed with 75 μl of pooled normal human serum in microtiter plates and then incubated at 37 °C for a period of 3 h. The test was performed in triplicate and the number of recovered bacteria was determined and graded. Resistance grading was defined from grade 1 to grade 6, with grade 6 (viable counts at 1, 2 and 3 h >100% and increasing throughout the 3-h period) considered to be the most serum resistant. Grades 5 and 6 were regarded as high-grade serum resistance.

### Mouse lethality experiment

Determination of the virulence of *K*. *pneumoniae* in mouse lethality tests and the median lethal dose (LD_50_) was performed as described previously^22^. In brief, female BALB/c mice (6–7 weeks old) were obtained from the National Laboratory Animal Center (NLAC) (Tainan, Taiwan). Mice were maintained under standard conditions of temperature, light and feeding according to NLAC guidelines and the Chi Mei Medical Center Animal Care and Use Committee approved-protocols (Permit Number: 100120771). Each dose was injected intraperitoneally with 0.1 mL of bacterial suspension into four mice. After 14 days, calculations were based on the number of survivors. The degree of virulence was read as highly virulent for an LD_50_ of <10^3^ CFU; moderate virulence for an LD_50_ of 10^4^–10^5^ CFU; low virulence for an LD_50_ of 10^6^–10^7^ CFU; and no virulence for an LD_50_ of >10^8^ CFU.

## Molecular genotyping of isolates

### Pulsed-field gel electrophoresis (PFGE)

PFGE, as a major strain typing method, was used to confirm genetic relatedness of isolates as described by Pfaller *et al*.^26^. Whole chromosomal DNA in agarose was digested with *XbaI* (Bio-Rad Laboratories, CA., USA), and the restriction fragments were separated in a CHEF Mapper XA System (Bio-Rad Laboratories, CA., USA). Cluster analysis was performed by the Dice similarity coefficients and unweighted-pair group matching algorithm (UPGMA) with a tolerance of 1.0% and 1.0% optimization using the BioNumerics program. All bands had to match exactly to classify isolates as indistinguishable. Isolates were designated nontypeable if repeated attempts to prepare DNA failed.

### Multilocus sequence typing (MLST)

MLST was performed with 7 housekeeping genes as previously described^27^. Multiple sequences alignment analysis was conducted by using Institute Pasteur (http://bigsdb.web.pasteur.fr/klebsiella/klebsiella.html).

### Data collection

The imaging studies and following main data relevant to patient characteristics were collected: gender, age, comorbidities, use of ventilator, CSF profiles, and random blood sugar obtained on the day of CSF collection. Outcome was described as in-hospital mortality within the same hospital episode.

### Statistical Analysis

The Fisher’s exact test was used for categorical variables and Student’s *t-*test was used for continuous variables, where appropriate. A two-tailed *p* value < 0.05 was considered statistically significant. All statistics were performed using Stata version 12.1 (Stata Press, College Station, TX, USA).

### Ethical approval

The study and waiver from the inform consent process were approved by the Institutional Review Board (IRB) of the Chi Mei Medical Center, Tainan city, Taiwan (IRB Serial number 10603-013).

## Results

### MIC

All 20 primary meningitis isolates were susceptible to cefotaxime and ceftazidime. Nine of 13 post-craniotomy meningitis isolates were resistant to cefotaxime and ceftazidime, but were all susceptible to meropenem, colistin and tigecycline. These 9 isolates were of multi-resistant phenotype. In contrast, all the 20 primary meningitis isolates were only resistant to ampicillin (Table 1).

**Table 1:** MIC profiles of cerebrospinal fluid *Klebsiella pneumoniae* isolates from post-craniotomy and primary meningitis.

MIC (μg/mL) of antibiotic compounds	Post-craniotomy meningitis (*n* = 13)	Primary meningitis (*n* = 20)	Total (*n* = 33)
Ampicillin			
MIC_50_	>16	>16	>16
MIC_90_	>16	>16	>16
MIC range	>16	>16	>16
Aztreonam			
MIC_50_	>16	≤1	≤1
MIC_90_	>16	≤1	>16
MIC range	≤1–>16	≤1	≤1–>16
Cefotaxime			
MIC_50_	>32	≤1	≤1
MIC_90_	>32	≤1	>32
MIC range	≤1–>32	≤1–2	≤1–>32
Ceftazidime			
MIC_50_	>16	≤1	≤1
MIC_90_	>16	≤1	>16
MIC range	≤1–>16	≤1	≤1–>16
Cefepime			
MIC_50_	2	≤1	≤1
MIC_90_	16	≤1	8
MIC range	≤1–>16	≤1	≤1–>16
Piperacillin-tazobactam			
MIC_50_	>64	≤4	≤4
MIC_90_	>64	8	>64
MIC range	≤4–>64	≤4–16	≤4–>64
Meropenem			
MIC_50_	≤0.25	≤0.25	≤0.25
MIC_90_	≤0.25	≤0.25	≤0.25
MIC range	≤0.25	≤0.25	≤0.25
Ciprofloxacin			
MIC_50_	2	≤0.06	≤0.06
MIC_90_	>2	0.12	2
MIC range	≤0.06–>2	≤0.06–025	≤0.06–>2
Colistin			
MIC_50_	1	1	1
MIC_90_	2	1	1
MIC range	≤0.5–2	≤0.5–1	≤0.5–2
Tigecycline			
MIC_50_	0.5	0.5	0.5
MIC_90_	1	1	1
MIC range	0.5–1	≤0.25–2	≤0.25–2

### ESBL and AmpC phenotypes

Resistance to cefotaxime and ceftazidime was found in 9 isolates. Among them, ESBL phenotype was detected in 7 isolates and AmpC phenotype was suspicious for 2 isolates (KP-004 and KP-042).

### ESBL and AmpC β-lactamase genes

PCR amplifications for β-lactamase genes were performed for all 33 isolates. ESBL genes were detected in 7 isolates (6 with *bla*_SHV-5_ and 1 with *bla*_TEM-1B_). AmpC genes were detected in 4 isolates (2 with *bla*_CMY-2_ and 2 with *bla*_DHA-1_), including 2 isolates with dual ESBL and AmpC genes (1 with *bla*_SHV-5_ plus *bla*_DHA-1_ and 1 with *bla*_TEM-1B_ plus *bla*_CMY-2_). KPC-2 gene was not identified. Overall, 9 of 13 (69.2%) post-craniotomy meningitis isolates produced ESBL and/or AmpC β-lactamases (Table 2).

**Table 2:** Epidemiology and virulence profiles of *Klebsiella pneumoniae* isolates from CSF of post-craniotomy and primary meningitis.

CSF Strains	PFGE	MLST	ESBL	AmpC	Virulence factors										LD_50_ (log)
					Bacterial size (μm)	HV (mm)	Capsule K	*rmpA*	*rmpA2*	*c-rmpA*	*Kfu*	*allS*	*aerobactin*	SR	
**Post-craniotomy meningitis** (***n*** = **13**)															
KP-004^a^	A	17	−	CMY-2	1.11 ± 0.21	<0.5	x	−	−	−	−	−	−	G2	>6.50
KP1050^b^	O	107	SHV-5	−	1.42 ± 0.16	<0.5	x	−	−	−	+	−	−	G6	>6.84
KP1051^b^	H1	17	SHV-5	−	1.44 ± 0.11	<0.5	x	−	−	−	−	−	−	G2	>6.72
KP1052^b^	N1	15	SHV-5	DHA-1	1.72 ± 0.07	<0.5	x	−	−	−	−	−	−	G6	>6.42
KP1053^b^	G	54	SHV-5	−	1.28 ± 0.15	<0.5	x	−	−	−	−	−	−	G6	>6.55
KP1054^b^	I1	86	SHV-5	−	2.98 ± 0.41	<0.5	2	+	+	−	−	−	+	G5	>6.88
KP-042^c^	unidentified	185	−	DHA-1	1.31 ± 0.18	<0.5	x	−	−	−	−	−	−	G4	>6.52
KP-023^c^	D	undefined	TEM-1B	CMY-2	0.92 ± 0.16	<0.5	x	−	−	−	+	−	−	G3	>6.36
KP-201^c^	E2	65	SHV-5	−	4.62 ± 0.65	>2	2	+	+	−	−	−	+	G6	4.13
KP-001^a^	J1	373	−	−	4.66 ± 0.66	>2	2	+	−	−	−	−	+	G3	5.83
KP-007^a^	S	661	−	**−**	1.04 ± 0.08	<0.5	x	−	−	−	−	−	−	G2	>6.84
KP-232^c^	E1	65	−	−	3.95 ± 0.46	>2	2	+	+	−	−	−	+	G5	5.05
KP-960^b^	N2	15	−	−	1.27 ± 0.17	<0.5	5	−	−	−	+	−	−	G6	>6.71
**Primary meningitis** (***n*** ** = 20**)															
KP-002^a^	C4	23	−	−	2.42 ± 0.33	>2	1	+	−	−	+	+	+	G5	4.83
KP-003^a^	C5	23	−	−	10.73 ± 1.11	>2	1	+	+	−	+	+	+	G6	4.50
KP-005^a^	M	268	−	−	7.24 ± 0.76	>2	20	+	+	−	−	−	+	G5	>6.49
KP-006^a^	Q	65	−	−	3.67 ± 0.42	0.5–1	2	+	+	−	−	−	+	G6	4.41
KP-064^c^	R1	218	−	−	3.74 ± 0.49	>2	57	+	+	−	−	−	+	G2	>6.45
KP-088^c^	P2	373	−	−	4.77 ± 0.46	>2	2	+	+	−	−	−	+	G6	1.59
KP-477^c^	C3	23	−	−	7.75 ± 1.37	>2	1	+	+	+	+	+	+	G5	4.41
KP-600^c^	C1	23	−	−	2.33 ± 0.24	<0.5	x	−	−	−	−	−	−	G2	>6.45
KP-777^c^	C2	23*	−	−	5.30 ± 0.71	>2	1	+	+	+	+	+	+	G6	4.98
KP-164^c^	R1	218	−	−	3.04 ± 0.48	>2	57	+	+	−	−	−	−	G3	>6.15
KP-552^b^	B	23	−	−	7.05 ± 0.72	>2	1	+	+	−	+	+	+	G5	5.35
KP-780^b^	P1	373	−	−	6.92 ± 0.53	>2	2	+	+	−	−	−	+	G6	>6.44
KP-783^b^	P1	373	−	−	3.06 ± 0.43	>2	2	+	+	−	−	−	+	G6	5.4
KP-957^b^	K	65	−	−	4.56 ± 0.56	>2	2	+	+	−	−	−	+	G6	3.94
KP-958^b^	I2	86*	−	−	3.45 ± 0.49	>2	2	+	+	−	−	−	+	G5	1.56
KP-959^b^	unidentified	1049	−	−	4.71 ± 0.89	>2	5	+	+	−	+	-	+	G6	>6.50
KP-961^b^	J2	373	−	−	4.58 ± 0.39	>2	2	+	+	−	−	−	+	G6	3.77
KP-962^b^	F	1544	−	−	5.09 ± 1.06	0.5–1	20	+	+	−	+	−	+	G6	5.14
KP-963^b^	H2	29	−	−	4.51 ± 0.53	>2	54	+	+	−	−	−	+	G6	5.41
KP-966^b^	L	592	−	−	2.75 ± 0.45	0.5–1	57	+	−	−	−	−	+	G2	>6.59

NOTE. CSF, cerebrospinal fluid; PFGE, Pulsed-field gel electrophoresis; MLST, multilocus sequence typing (*one nucleotide change); SR, serum resistance (G: grade); ESBL, extended-spectrum β-lactamase; HV, hypermucoviscosity phenotype. x, unidentified, non-virulent capsule K types (i.e., not for K1, K2, K5, K20, K54, K57 types). ^a^From Taipei Veterans General Hospital in Taipei City, northern Taiwan, 2003–2006 (*n* = 7); ^b^from Chi-Mei Medical Center in Tainan City, southern Taiwan, 2005–2013 (*n* = 16); ^c^from National Cheng-Kung University Hospital in Tainan City, southern Taiwan, 1999–2007 (*n* = 10).

### Hypermucoviscosity (HV) phenotype

The prevalence of HV phenotype was 66.7% (22/33) of *K*. *pneumoniae* CSF isolates in general, and was specifically in the subgroups of the isolates as follows: (1), 95.0% (19/20) of primary meningitis isolates; (2), 23.1% (3/13) of post-craniotomy isolates and (3), 11.1% (1/9) of ESBL/AmpC-producing isolates (Table 2). Primary meningitis isolates were significantly more hypermucoviscous than post-craniotomy meningitis isolates (*p* < 0.0001), in accordance with our hypothesis that hvKP was the main etiology of primary KP meningitis, while cKP was mainly responsible for post-craniotomy meningitis (Table 3). The cKP group has higher prevalence of ESBL/AmpC production than hvKP group (9/11 vs. 0/22, *p* < 0.0001, Table 2).

**Table 3:** Different virulence profiles between cerebrospinal fluid *Klebsiella pneumoniae* (KP) isolates from post-craniotomy and primary meningitis.

Cerebrospinal fluid KP isolates (*n* = 33)	Post-craniotomy meningitis (*n* = 13)	Primary meningitis (n = 20)	*p*
ESBL/AmpC genotypes	9	0	<0.0001*
Bacterial (capsule) size			
>2 mm (*n* = 24)	4	20	<0.0001*
Hypermucoviscosity			
Phenotype (*n* = 22)	3	19	<0.0001*
*rmpA* (*n* = 23)	4	19	0.0002*
*rmpA2* (*n* = 20)	3	17	0.0007*
*c-rmpA* (*n* = 2)	0	2	0.5076
Capsule K serotype			
Virulent K types (K1, K2, K5, K20, K54, K57)	5	19	0.0007*
K1/K2	4	12	0.1571
K1	0	5	0.1310
K2	4	7	1.0000
K5	1	1	1.0000
K20	0	2	0.5076
K54	0	1	1.0000
K57	0	3	0.2614
*Kfu* (*n* = 10)	3	7	0.7006
*allS* (*n* = 5)	0	5	0.1310
Aerobactin gene (*n* = 22)	4	18	0.0007*
Serum resistance			
Grades 5–6 (*n* = 23)	7	16	0.1393
Grades 3–4	3	1	0.2757
Grades 1–2	3	3	0.6588
LD_50_ (log)			
>6 (*n* = 17)	10	7	0.0324*
4–6	3	9	0.2775
<4	0	4	0.1359

### Bacterial (capsule) size

By measuring the short transverse diameter, mean bacterial size of each bacterium ranged from 0.92 ± 0.16 μm to 10.73 ± 1.11 μm. All 20 isolates recovered from primary meningitis had mean bacterial sizes of more than 2 μm. All nine strains with a mean bacterial size of <2 μm were post-craniotomy meningitis isolates, which were significantly different to primary meningitis isolates (*p* < 0.0001). In addition, only two of the nine isolates with ESBL/AmpC β-lactamase production had a mean bacterial size more than 2 μm (Tables 2 and 3).

The HV phenotype was highly prevalent in the isolates with mean bacterial size of >2 mm (91.7%, 22/24), whereas none of the nine isolates with mean capsule size of <2 mm had the HV phenotype (*p* < 0.0001).

### Capsule K genotype

The common liver abscess-associated capsule serotypes were identified in 24 (72.7%) isolates by PCR methods, including capsule K2 (11 strains), K1 (5 strains), K57 (3 strains), 2 strains each for K5 and K20, and one K54 respectively. These virulent K serotypes were highly prevalent in primary meningitis isolates than in post-craniotomy isolates (19/20 vs. 5/13, *p* = 0.0007). However, specific K serotype (such as K1 or K2) was not statistically different between the two groups (Table 3).

Meanwhile, the isolates with virulent K serotypes had higher prevalence of >2 μm bacterial size (23/24 vs. 1/9, *p* < 0.0001), higher proportion of HV phenotype (22/24 vs 0/9, *p* < 0.0001), and lower prevalence of ESBL/AmpC production (2/24 vs. 7/9, *p* = 0.0003) than those with undefined capsule K serotypes (Table 2).

### Prevalence of virulence-associated genes

Among the isolates of *K*. *pneumoniae* recovered from CSF, the prevalence of *rmpA* was 69.7% (23/33); *rmpA2*, 60.6% (20/33); *c-rmpA2*, 6.1% (2/33); *kfu*, 30.3%, (10/33); *alls*, 15.6% (5/33); and aerobactin gene, 66.7% (22/33). In general, the *rmpA*, *rmpA2* and aerobactin gene were highly prevalent in primary meningitis isolate than in post-craniotomy isolates (Table 3).

The HV phenotype was highly prevalent in those with *rmpA*-positive vs. *rmpA*-negative isolates (22/23 vs. 0/10, *p* < 0.0001), with *rmpA2*-positive vs. *rmpA2*-negative isolates (19/20 vs. 3/13, *p* < 0.0001) and with aerobactin-positive vs. aerobactin-negative isolates (21/22 vs. 1/11, *p* < 0.0001). In similar, the isolates positive for *rmpA*, *rmpA2* and aerobactin gene had larger bacterial size (≥2 μm) and were highly prevalent in the isolates with virulent K serotypes (such as K1, K2, K5, K20, K54 and K57).

### Serum resistance

In general, the distribution of isolates in the grading of serum resistance were 23 strains in grades 5 and 6; grade 4 (1 strain), grade 3 (3 strains); and grade 2 (6 strains) (Table 3). Unexpectedly, the prevalence of high serum resistance (grades 5 and 6) could not reach statistically significant difference between primary meningitis isolates and post-craniotomy isolates (16/20 vs. 7/13, *p* = 0.1393), which was distinct from the above-mentioned virulence factors between groups (Table 3).

However, the 23 isolates with high serum resistance (grades 5–6) were still significantly associated with virulence K serotypes (*n* = 20, *p* = 0.0105), HV phenotype (*n* = 18, *p* = 0.0494), *rmpA* (*n* = 19, *p* = 0.035), *rmpA*2 (*n* = 19, *p* = 0.0007) and aerobactin gene (*n* = 19, *p* = 0.0059), in comparison to isolates with lower grade serum resistance (grades 1–4).

### Mouse lethality experiments

LD_50_ > 10^6^ (low virulence) was found in 10 of 13 post-craniotomy isolates, but in 7 of 20 primary meningitis isolates (*p* = 0.0324). The prevalence of virulence factors were significantly higher in isolates with LD_50_ < 10^6^ (high virulence) than those with low virulence (LD_50_ > 10^6^), including mean bacterial size >2 μm (16/16 vs. 8/17, *p* = 0.0009), HV phenotype (16/16 vs. 6/17, *p* < 0.0001), *rmpA* (16/16 vs. 7/17, *p* = 0.0003), *rmpA2* (14/16 vs. 6/17, *p* = 0.0039), aerobactin gene (16/16 vs. 6/17, p < 0.0001), virulent K serotypes (16/16 vs. 8/17, *p* = 0.0009) and high-grade serum resistance (15/16 vs. 8/17, *p* = 0.0066).

### PFGE

**Figure 1:**
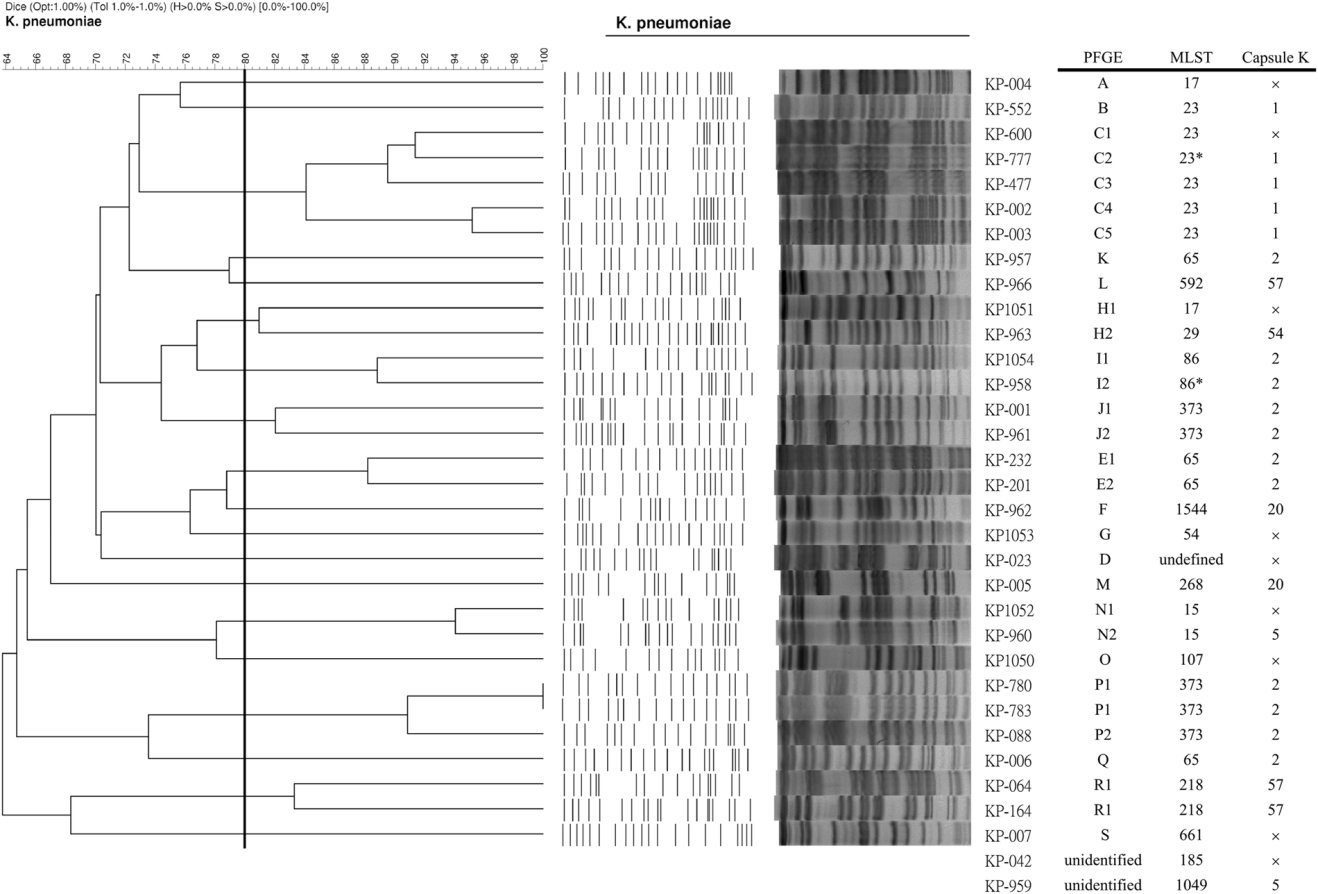
Fingerprinting profiles (A to S) of XbaI-digested genomic DNA using the Dice coefficient and UPGMA for *Klebsiella pneumoniae* cerebrospinal fluid isolates collected in Taiwan. Eight pulsotypes (C, E, H, I, J, N, P, and R) show a Dice similarity coefficient of more than 80%. Corresponding MLST (multilocus sequence typing) and capsule K serotype of each isolate were presented. *One nucleotide change; x, unidentified, non-virulent capsule K types, i.e., not for K1, K2, K5, K20, K54, K57 types.

Two isolates were nontypeable. The remaining 31 isolates were classified into 19 major pulsotypes (A~S types) by using the Dice coefficient and UPGMA algorithm (Fig. 1). Among them, 8 pulsotypes with a similarity coefficient of more than 80% included 5 isolates in pulsotype C (C1-C5), 3 isolates in pulsotype P (P1-P2) and 2 isolates each in pulsotype E (E1-E2), pulsotype H (H1-H2), pulsotype I (I1-I2), pulsotype J (J1-J2), pulsotype N (N1-N2) and pulsotype R (R1-R2). In general, no major single clone (indistinguishable or >95% similarity) was broadly disseminated among the *K*. *pneumoniae* strains that caused meningitis in Taiwan.

### MLST

From all isolates performing MLST, 6 strains were ST23 (including one ST23-like variant), 5 strains were ST373, 4 strains were ST65, 2 strains were ST86 (including one ST86-like variant), ST15, ST17 and ST218 respectively. Others were one each for ST29, ST54, ST107, ST185, ST268, ST592, ST661, ST1049, ST1544 and unidentified ST (strain KP-023).

The association between genotyping, sequence type and K capsule serotypes of *K*. *pneumoniae* meningitis isolates were summarized in Fig. 1. Notably, the 5 primary meningitis isolates of pulsotype C (C1-C5) were capsule serotype K1, including ST23 in 4 strains and ST 23-like in 1 strain. Five ST373 strains belonged to pulsotypes J and P. Isolates of ST373, ST65 and ST86 belonged to capsule serotype K2. Two isolates of ST218 and 1 isolate of ST29 belonged to capsule serotype K57 and K54 respectively.

### Demographic data and clinical outcomes

**Figure 2:**
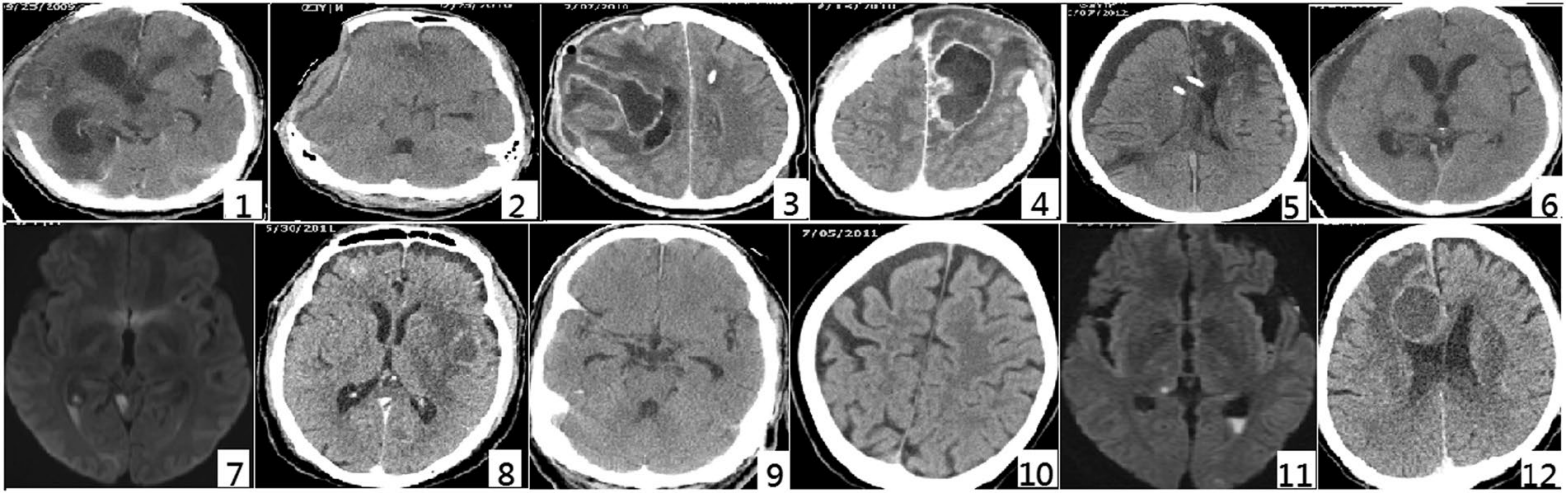
Brain imaging studies of patients with *Klebsiella pneumoniae* meningitis from post-craniotomy meningitis groups (no. 1–6) and primary meningitis group (no. 7–12). No. 1, strain KP1050, hydrocephalus (computed tomography, CT); No. 2, strain KP1051, brain edema with compression effect (CT); No. 3, strain KP1052, brain abscess (CT); No. 4, strain KP1053, brain abscess (CT); No. 5, strain KP1054, subdural effusion (CT); No. 6, strain KP-960, subdural effusion (CT); No. 7, strain KP-958, ventriculitis (Magnetic Resonance Imaging, MRI); No. 8, strain KP-959, brain abscess (CT); No. 9, strain KP-961, hydrocephalus; No. 10, KP-962, essentially intact in brain parenchyma (CT); No. 11, strain KP-963, ventriculitis (MRI); and No. 12, strain KP-966, brain abscess (CT).

The 33 *K*. *pneumoniae* isolates from CSF basically of primary meningitis or post-craniotomy meningitis were collected from 1999 to 2013. However, detailed clinical data could be retrieved for the 12 patients from 2009 to 2013 (Table 4). The reasons of craniotomy were mainly to remove hematoma of various causes. Five of six patients in this group underwent craniectomy and two patients developed brain abscess (Fig. 2). The main comorbidities of patients with primary meningitis were diabetes mellitus and alcoholic liver cirrhosis (Table 4). Two patients in this group developed brain abscess and two patients developed ventriculitis (Fig. 2). Only one patient died during the hospital episode. There were no statistically significant differences in the demographic data, CSF profiles and clinical outcomes between the two groups of *K*. *pneumoniae* meningitis.

**Table 4:** Clinical profiles and outcome of *Klebsiella pneumoniae* isolates from CSF of post-craniotomy and primary meningitis.

Strain^a^	Age (yr)	Sex	Comorbidity	Ventilator use	Sugar^b^ (mg/dl)	CSF profiles						Outcome
						RBC (per μl)	WBC (per μl)	Neutrophil (%)	Lymphocyte (%)	Sugar (mg/dl)	Protein (mg/dL)	
**Post-craniotomy meningitis**												
KP1050	56	M	Head injury	Nil	149	5	18	72	28	54	108.0	Survived
KP1051	23	M	SAH	Nil	Nil	1030	133	60	40	57	57.5	Survived
KP1052	59	M	ICH	Yes	Nil	1250	2016	92	8	23	873.5	Survived
KP1053	61	M	AVM rupture	Yes	Nil	200	75900	100	0	<1	592	Survived
KP1054	51	F	AVM rupture	Nil	Nil	110	1177	72	28	<1	206.7	Survived
KP-960	56	M	Head injury	Nil	Nil	0	6	50	50	1	412.0	Survived
**Primary meningitis**												
KP-958	48	M	DM, ALC	Nil	342	760	1632	97	3	118	>500	Survived
KP-959	62	M	ALC	Yes	Nil	24500	94600	65	35	1	>500	Died
KP-961	55	M	DM	Nil	489	576	12672	97	3	219	325	Survived
KP-962	68	F	DM	Nil	405	30	372	71	29	205	173.3	Survived
KP-963	50	M	DM	Nil	430	190	5712	97	3	241	>500	Survived
KP-966	62	M	ALC	Yes	157	27000	62700	72	28	<1	>500	Survived
*p*	0.390	1.000	NA	1.000	NA	0.176	0.522	0.345	0.345	NA	NA	1.000

Note CSF, cerebrospinal fluid; M, male; F, female; SAH, subarachnoid hemorrhage related to a brain aneurysm; ICH, intracerebral hemorrhage; AVM, arteriovenous malformation; DM, diabetes mellitus; ALC, alcoholic liver cirrhosis; RBC, red blood cell count (normal, <5/μl); WBC, white blood cell count (normal, <5/μl); NA, not applicable; ^a^from Chi-Mei Medical Center in Tainan City, southern Taiwan, 2009–2013 (*n* = 12); ^b^random blood sugar obtained on the day of CSF collection.

## Discussion

The study investigated the antibiotic resistance profiles, β-lactamase genes, various hypervirulence determinants, mouse lethality, as well as the epidemiology of genomic macrorestriction patterns and sequence types of 33 *K*. *pneumoniae* isolates that were capable of invading the central nervous system to cause primary meningitis (*n* = 20) or post-craniotomy meningitis (*n* = 13). Generally consistent with our hypothesis, primary meningitis isolates were more susceptible to third- and fourth-generation cephalosporins, were more hypervirulent (hvKP) and had higher mouse lethality capability than post-craniotomy meningitis isolates (mostly cKP). Exceptionally, serum resistance did not reach statistically significant difference between groups, suggesting that high-grade serum resistance (*n* = 23) is the major common virulence determinant of *K*. *pneumoniae* isolates with capability to invade the central nervous system, regardless of primary or post-craniotomy meningitis.

The post-craniotomy isolates had significantly higher carriage rate of ESBL/AmpC genes (69.2%) than primary meningitis isolates did (0%), suggesting that it is appropriate for meropenem but not for broad-spectrum cephalosporin in the empirical therapy of post-craniotomy meningitis. Chang *et al*. reported data of 49 adult *K*. *pneumoniae* meningitis cases (36 spontaneous meningitis and 13 post-neurosurgical meningitis), collected over a period of 11 years (2000–2010) in Taiwan^28^. Data on antibiotic resistance of these isolates also showed none of 36 spontaneous meningitis isolates but only 2 of 13 (15.4%) post- neurosurgical meningitis isolates were resistant to third- and fourth-generation cephalosporins and one of the strains was ESBL-producing^28^. Thus, it is important to monitor the trend of antibiotic resistance in *K*. *pneumoniae* CSF strains for the empirical antibiotic strategy of meningitis.

With regard to specific virulence factors, primary meningitis isolates were more virulent than post-craniotomy isolates in term of the expression of larger bacterial size, HV phenotype, *rmpA*, *rmpA*2, aerobactin gene, virulent capsule K serotypes (K1, K2, K5, K20, K54, and K57), and high mouse lethality (LD_50_ < 10^6^). Capsular K2 serotype was the most common serotype observed (33.3%). Most hvKP isolates were significantly associated with the above-mentioned virulence factors and exhibited high-grade serum resistance as well as high mouse lethality. Furthermore, aerobactin is one of the siderophore systems, which mediate acquisition of iron to help virulent bacteria to overcome iron starvation, while bacteria invade and proliferate in the human systems^14, 24, 25^. In general, the current data support our initial hypothesis. The presence of these virulent genes and characters of primary meningitis hvKP isolates might assist in their capability to invade CSF space through intact central nervous system barrier to cause infection, whereas the less virulent cKP might still be able to cross the CSF space through disruption of central nervous system barrier, probably with the aid of the mechanism of serum resistance. We found that high-grade serum resistance occurred in 7 of 13 (53.8%) post-craniotomy isolates and in 16 of 20 (80%) in primary meningitis isolates, without reaching significant difference (*p* = 0.139). We acknowledge that high serum resistance is the major common virulence determinant of hvKP and cKP isolates contributing to invade the central nervous system, regardless of primary or post-craniotomy status.

With regard to molecular epidemiology, no major genomic clone or sequence type broadly existed among the 33 *K*. *pneumoniae* CSF isolates. However, there were 5 primary meningitis isolates of pulsotype C (C1-C5) belonging to ST 23 or ST23-like sequence type. The ST23 has been recognized as the most prevalent MLST type of capsule serotype K1 *K*. *pneumoniae* isolates from liver abscess in Taiwan^29^. The ST65, ST86, ST373 and ST375 (not detected in the study) have been the major clones associated with K2 *K*. *pneumoniae* liver abscesses in Taiwan^29, 30^. In addition, ST65, ST65-like and ST86-like MLST types were most predominant among K2 serotype isolates of the community-acquired infection cases from Singapore, Hong Kong and China^31, 32^.

ST218 has been reported in capsule serotype K57 liver abscess isolates in Taiwan^30^. ST29 K54 ESBL-producing *K*. *pneumoniae* with HV phenotype was detected in Hunan, China^33^. We previously reported on a mycotic aneurysm caused by hypermucoviscous ST29 K54 *K*. *pneumoniae* in Taiwan^34^. Furthermore, the ST29 was the predominant ST of carbapenem-resistant *K*. *pneumoniae* isolates in Saudi Arabia^35^. The ST15, ST17, ST54, and ST107 isolates producing ESBL, DHA-1, KPC-2 or NDM-1metallo-β-lactamase have been found in Taiwan, China and other various geographic areas in the world^36–42^. Importantly, 5 of 9 ESBL/AmpC producers in our current study belonged to these international clones, which often exhibited high-grade serum resistance. In addition to our recent report on the bacteremic isolates^43^, we continuously highlight the importance of monitoring the emergence of hypervirulence in the ESBL/AmpC-producing *K*. *pneumoniae*, particularly in the CSF isolates.

Although the *K*. *pneumoniae* isolates had different virulence mechanisms between the groups of primary and post-craniotomy meningitis, they did not result in significantly different clinical features and outcomes among limited patients between groups. The reasons could be explained by the effectiveness of BSC and meropenem against the isolates causing primary and post-craniotomy meningitis respectively. However, continuous monitoring for the emerging resistance profiles of *K*. *pneumoniae* between different groups of meningitis is clinically important.

## Conclusion

Primary *K*. *pneumoniae* meningitis isolates had hypervirulence profiles of virulent capsule K serotypes, larger capsule size, HV phenotype, *rmpA*, *rmpA2*, aerobactin gene, high serum resistance and high capability of mouse lethality. Post-craniotomy *K*. *pneumoniae* meningitis isolates had relatively low virulence profiles and exhibited low mouse lethality, but still had high serum resistance which supported their capabilities to invade the central nervous system. The MLST international clones (ST15, ST17, ST54, and ST107) have been found in the post-craniotomy meningitis isolates in Taiwan, which could produce ESBL/AmpC β-lactamases. Therefore, physicians should be aware the emerging trend of antibiotic resistance in the empirical treatment for *K*. *pneumoniae*, particularly in post-craniotomy meningitis.
